# Prevalence of *Treponema Pallidum* Antibody among Volunteer Blood Donors in China

**DOI:** 10.1155/2022/1668703

**Published:** 2022-08-08

**Authors:** Xiaofan Zheng, Wei Ding, Xia Ling, Jie Shi, Jie Dong, Guangshu Yu, Yan Chen, Rui Li, Lihong Xu, Xiaotao Li, Hong Zhu, Faming Zhu, Wei Hu

**Affiliations:** ^1^Blood Center of Zhejiang Province, Hangzhou, Zhejiang 310052, China; ^2^Key Laboratory of Blood Safety Research of Zhejiang Province, Hangzhou, Zhejiang 310052, China

## Abstract

**Background:**

Infection with syphilis is still a major public health problem. The precise data for syphilis seroprevalence in the populations will help to develop a strategy for prevention and treatment of it. However, the data for syphilis prevalence in continuous years among volunteer blood donors in China is rare.

**Methods:**

A retrospective study for *Treponema pallidum* (TP) antibody in blood donors was conducted from January 2010 to December 2019 at the Blood Center of Zhejiang Province, China. TP antibody was detected with two different reagents using enzyme-linked immunosorbent assay and the only sample which was reactive in the two reagents was defined as seropositive.

**Results:**

A total of 992,646 volunteer blood donors were analyzed and the positive rate of TP antibody in the blood donors was 0.43%. From 2010 to 2019, the positive rates of TP antibody were 0.53%, 0.51%, 0.51%, 0.43%, 0.36%, 0.18%, 0.11%, 0.12%, 0.11%, and 0.10%, respectively. The positive rates of TP antibody were significantly different among blood donor age group (*p* < 0.001), with the highest positive rate in 45–54-years-old group (0.93%). The positive rates of TP antibody in male and female blood donors were 0.44% and 0.41%, respectively. The positive rate was 0.57% among the first-time blood donors, which was significantly higher than that of the repeat blood donors (0.17%). The positive rate of TP antibody in blood donors decreased gradually with the increase of educational level.

**Conclusion:**

The syphilis seroprevalence is low in the blood donors of the Hangzhou area, and the positive rate of blood donors is associated with age, educational level, and times of blood donation. Increasing the number of repeat blood donations is helpful to improve blood safety.

## 1. Background

Syphilis, an infectious disease with a long history, is still a severe challenge to public health worldwide. The World Health Organization (WHO) estimates that there are 12 million new cases of syphilis each year, with more than 90% occurring in developing nations [1, 2]. Furthermore, there has been a resurgence of syphilis both in developed and developing countries since the late 1990s [3–5]. Since the founding of the People's Republic of China in 1949, owing to the relentless efforts of the government, syphilis along with other sexually transmitted diseases (STDs) were virtually eliminated in mainland China in the 1960s [6]. However, since the reform and opening up of the 1980s, a great comeback of syphilis has been seen in China [7, 8]. In recent years, owing to interventions by health authorities, the increased rate of the reported incidence of syphilis in China has been slowing down, but it remains high and keeps growing steadily [9–11]. It indicates that the epidemic of syphilis infection is still threatening public health in China.

The pathogen of syphilis is *Treponema pallidum* (TP), a member of the treponema genus of the spirochetes family in the broad category of bacteria. The most common form of syphilis transmission is through sexual intercourse, as well as through mother-to-infant and blood transfusion. In order to prevent and reduce the risk of transfusion infectious syphilis disease, screening for TP antibodies in blood donors is routinely carried out in China [12, 13]. The positive of TP antibody test not only means that the blood donated has a possibility to transmit syphilis through transfusion but also implies that the donor may have other kinds of STDs, such as human immunodeficiency virus (HIV), which may pose a severe threat to blood safety [14, 15].

To evaluate the prevalence of TP antibodies among volunteer blood donors in Hangzhou, Zhejiang Province, China in continuous years, we retrospectively analyzed the results of TP antibody test in blood donors from 2010 to 2019. We also analyzed the prevalence and distribution of TP antibodies among different groups of blood donors characterized by age, gender, educational level, and number of blood donations.

## 2. Methods

### 2.1. Study Population

From January 2010 to December 2019, all volunteer blood donors in the Blood Center of Zhejiang Province, China were analyzed. The donor's medical record card number was used as the unique flag of the identification of the blood donors. The information and lab results will be used for research purposes anonymously, and this study was approved by the Ethical Scientific Committee of the Blood Center of Zhejiang Province, China.

Prior to blood donation, all potential donors needed to sign a donation registration form, complete a health history questionnaire, and undergo a health examination according to the blood donor selection criteria issued by the Chinese National Health Commission. Then, the donors will undergo predonation testing, which includes hemoglobin (Hb), ABO blood group, alanine aminotransferase (ALT), and hepatitis B surface antigen (HBsAg) rapid test (colloidal gold strip method, InTec Xinchuang Co., Ltd., Xiamen, China). Since 2015, HBsAg/TP antibody together rapid test was conducted. If all these results meet the criteria in China, then the donor can donate blood and the blood sample is collected for laboratory testing. For each blood donor, sociodemographic data including sex, age, and educational level were collected in the registration form and then registered in the blood management information system of the Blood Center of Zhejiang Province, China.

### 2.2. Syphilis Testing

TP antibodies were detected using the enzyme-linked immunosorbent assay (ELISA) method. According to China's National Standards, the TP antibody test is done twice using two different ELISA commercial kits provided by two different manufacturers (Wantai Pharmaceutical Co., Ltd., Beijing, China, and InTec Xinchuang Co., Ltd., Xiamen, China). All procedures were performed according to the manufacturer's instruction, in the Microlab Star 8CH and FAME 24/30 Automatic Enzyme Immunoassay Analyzer (Hamilton, Bonaduz, Switzerland). All initial reactive samples were retested once using the same ELISA assay. The nonreactive sample in both reagents was defined as negative. The reactive sample in any reagents was as reactive and the donated blood was discarded. However, only the test result of a sample was reactive to both of the two reagents, so it was regarded as seropositive in this study.

To assess the validity of this strategy, we collected partial samples with reactive results from dual ELISA test kits and samples with reactive results from single ELISA test kits for further TP antibody confirmatory test by the Treponema pallidum particle agglutination (TPPA) method (Fujirebio, Japan). In this pilot study, 151 blood samples with dual ELISA test kits reactive results were collected and retested. Among them, 137 (90.7%) were TPPA positive, and 14 (9.3%) were TPPA negative. Furthermore, 151 blood samples with a single ELISA test kit reactive results were tested by the TPPA method. Among them, 28 (18.5%) were TPPA positive, and 123 (81.5%) were TPPA negative. So we use the survey's data to assess the validity of this strategy: among the 992,646 volunteers who donated blood in 2010 to 2019, 4,316 blood donors were reactive in both ELISA test kits; we can infer that 401 (9.3%) blood donors were false positive; on the other hand, 1,671 blood donors were reactive in a single ELISA test kit; we can infer that 309 (18.5%) blood donors were false negative. This implies that, though a few positive samples may be missed, the positive rate of this strategy is close to the real situation.

### 2.3. Statistical Analysis

To calculate the prevalence of TP antibody year-by-year, the following strategy was used. If different blood donations by the same donor happen in the same calendar year, then it is calculated as one blood donation. All data of the blood donors were collected and stored in the blood management information system of the Blood Center of Zhejiang Province, China. Statistical parameters analysis was performed with the SPSS 24.0 version software, and comparison for the rate of difference groups was performed with the chi-square test. The proportions were calculated for the different prevalence types and the results were presented with a bilateral confidence interval of 95% using the Wilson score method with continuity correction. The multivariate logistic regression analyses were performed to determine potential predictors of TP seropositivity. The level of significance for each analysis was set at 0.05.

## 3. Results

### 3.1. Comparison of the Epidemic Trend of TP Antibody among Blood Donors from 2010 to 2019

**Figure 1:**
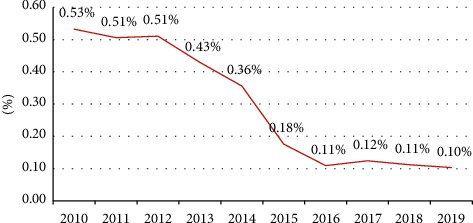
TP positive rate of blood donor in Hangzhou from 2010 to 2019.

Data for analysis were included a total of 1,689,699 valid blood donations, collected from 992,646 donors from 2010 to 2019. About 60% of the donors were male and 40% were female. Other data for sex, age, marital status, educational level, and donation times are listed in Table 1. Among the 992,646 volunteers who donated blood in 2010 to 2019, 4,316 blood donors were TP antibody positive, indicating an overall prevalence of 0.43%. From 2010 to 2019, the positive rates of TP antibody were 0.53%, 0.51%, 0.51%, 0.43%, 0.36%, 0.18%, 0.11%, 0.12%, 0.11%, and 0.10%, respectively. The positive rate of TP antibody was relatively constant after a sharp decrease from 2013 to 2016. The differences of TP antibody-positive rates among the calendar years were statistically significant (*χ*^2^ = 1599.70, *p* < 0.001) (Figure 1).

### 3.2. Prevalence of TP Antibody in Different Age Groups of Blood Donors from 2010 to 2019

**Figure 2:**
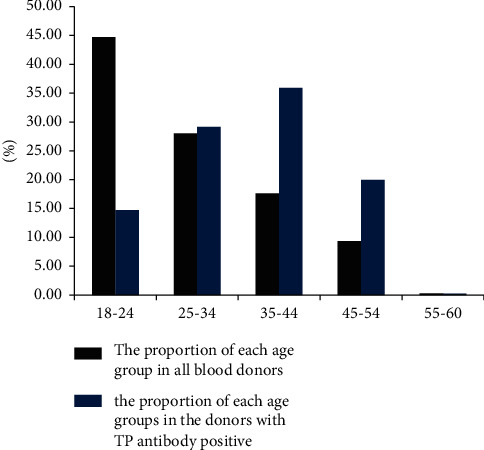
Distribution of blood donors by age groups. Each black row represents the proportion of the number of donors in the age group among the 992,646 blood donors. Each blue row represents the percentage of TP antibody positive in the age group among the 4,316 TP antibody-positive blood donors.

According to the age characteristics of blood donors, they were divided into five age groups (Table 1 and Figure 2). The proportion of each age group in all blood donors was 44.70%, 28.03%, 17.66%, 9.32%, and 0.29%, respectively, while the percentage of each age group in the donors with TP antibody positive was 14.76%, 29.17%, 35.89%, 19.90%, and 0.28%, respectively (Figure 2). The relational ratio (percentage of TP antibody positive divide by proportion of blood donors in each age group) in the five age groups were 0.33, 1.04, 2.03, 2.14, and 0.97, respectively, which indicated that a higher percentage of TP antibodies existed in the 35–44 and 45–55 age groups.

### 3.3. Prevalence Rates of the TP Antibody in the Donors Characterized by Gender, Age, Number of Donations, and Educational Level

The prevalence of TP antibody was 0.44% and 0.41% in the men's and women's groups, respectively. There was no significant difference between these two groups (Table 1). However, there was an upward trend in the prevalence of TP antibodies with the increase of age, except for the 56–60 group. The TP antibody positive rates of 18–24-year-old group and 56–60–year-old group were relatively lower, whereas the positive rate of 45–54-year-old group was the highest (0.93%) (Table 1). The difference in TP antibody prevalence in all age groups was statistically significant. The positive rate of TP antibody was 0.57% in the first-time blood donors, which was significantly higher than that in repeat blood donors (Table 1). The results also showed that the positive rate of TP antibody decreased gradually with the increase of the educational level (Table 1).

### 3.4. Logistic Regression Analysis

Multivariate logistic regression analyses were performed to determine potential predictors of TP seropositivity (Table 2). It showed that with the increase of age, male increased the risk factors of syphilis infection, and the increase of blood donations decreased the positive rate of TP antibody. Compared with high school education, the positive rate of TP antibody in primary and junior high school increased, but the positive rate of TP antibody in college degree, bachelor's, and postgraduate degrees was lower than that in high school education.

## 4. Discussion

According to the WHO, about 12 million individuals worldwide are newly infected with TP every year, and it has reported an infection rate of nearly 1% in the Chinese population [15]. However, the infection rate of TP in blood donors is different from that in the general population [16, 17]. Generally, the infection rate of blood donors is lower than that in the general population. The volunteer blood donors are considered as a selective population [16–18], since they are prescreened for previous diseases and sexual behavior, both by the detailed questionnaire and rapid screening of TP antibody before donation. During the process of blood donor interview and consultation, some infected individuals may not participate in blood donation by self-exclusion, and some infected individuals may be permanently deferred by health examination and rapid screening of TP antibody before blood donation. In addition, there is repeated blood donation among blood donors who have been tested negative many times. Therefore, the TP infection rate of blood donors is much less than that in the general population [16, 17]. The TP antibody-positive rate of blood donors in Hangzhou from 2010 to 2019 in this study was lower than that reported in some blood donor centers in China [13, 19], and also lower than the infection rate of the general population and high-risk behavior individuals [10, 20, 21]. Liu et al. reported that the seroprevalence of syphilis was 1.47% and 1.46 in 2016 and 2017 [13], and Song et al. reported that the overall prevalence of syphilis seropositivity was 0.70% among blood donors in Western China [19], while Shi et al. reported that the TP infection rate of the general population was 1.2% in a rural area of southwestern China [21]. However, the prevalence of TP in blood donors in Hangzhou is higher than that of blood donors in the USA (0.16%) and lower than that of blood donors in Colombia (1.86%) [17, 22]. In this study, the positive rate of TP antibody decreased significantly from 2013 to 2016, which was mainly related to a change in the blood screening strategy of blood transfusion services. In 2013, HBsAg/TP antibody together with a colloidal gold strip rapid test was first used in Jiande Blood Station of Hangzhou area to screen blood donors before donation. The positive rate of TP antibody decreased from 0.870% in 2012 to 0.037% in 2013. This screening method was gradually popularized in other collection sites in the Hangzhou area and was fully implemented by 2015, thus greatly reducing the participation of TP antibody-positive people in blood donation. Therefore, the overall positive rate after the implementation of rapid testing showed a significant decline from 2013 to 2016.

The numbers of the blood donations were used and analyzed in some previous studies for the TP antibody-positive rate in blood donors [13, 23]. However, the annual repeat blood donor was treated as one individual in this study, which can avoid the influence of the number of repeat blood donations on the positive rate calculation. In the age distribution, the 18–34 year-old-group were the majority of blood donors (about 67%). The proportion of those participating in blood donation gradually decreased with the increase of age, which may be related to the health status and consciousness of blood donation of individuals in different age groups. The positive rate of TP antibody in the 18–24–years-old group and the 55–60 years-old group was significantly lower than that in the 25–54-year-old group, which was similar to the previous reports [24]. The 18– 24 year-old group was mainly composed of college students in our study, who had a lower risk of sexually transmitted syphilis, while all blood donors in the 55 to 60 year-old group were repeated blood donors, so the positive rate of TP antibody in these groups was relatively low. Syphilis transmission is mainly through sexual contact. The risk of transmission is related to protective measures, health knowledge, and so on. The results showed that the positive rate of TP antibody decreased with the increase of educational level. This phenomenon may be related to the correlation between the individual's educational level and the awareness rate of health prevention knowledge. When the educational level is relatively high, the awareness rate of health knowledge is relatively high and the risk of infection is low [25, 26]. In this study, we found that the positive rate of TP antibody in the first blood donors was significantly higher than that in the repeat blood donors. The results are consistent with those reported in previous studies [27, 28]. Therefore, the risk of transmissible diseases is much higher in the first blood donors than in the repeat blood donors. From the blood safety point of view, blood services should recruit as many nonremunerated blood donors as possible and keep them well so as to increase the number of repeat blood donors. This will improve blood safety.

However, there are some limitations in our study. One is that we did not do additional confirmatory tests for ELISA reactive samples. As mentioned before, we used reactive in both ELISA test kits as the indicator for TP antibody prevalence, which was also used by other researchers in previous studies [13, 28].

## 5. Conclusion

The data for syphilis prevalence in ten years among volunteer blood donors in China was obtained, which showed the low syphilis seroprevalence in blood donors of the Hangzhou area, China. However, the TP antibody-positive rate of blood donors is associated with age, educational level, and times of blood donation. Therefore, developing a strategy according to these factors will help to improve blood safety.

## Figures and Tables

**Table 1:** Prevalence of TP antibody in the donors by gender, age, number of donations, and educational level.

Variable	Number of donors	Number of seropositives	Prevalence (%)	95% CI of prevalence (%)	*p* value
Total	992646	4316	0.43	0.420–0.446	
*Gender*					

Men	595933	2683	0.44	0.431–0.465	*χ * ^2^ = 8.191, *p* > 0.05
Women	396713	1633	0.41	0.390–0.430	
*Age*					

18–24 years	443701	637	0.14	0.132–0.155	*χ * ^2^ = 2207.796, *p* < 0.001
25–34 years	278209	1259	0.45	0.426–0.475	
35–44 years	175293	1549	0.88	0.833–0.919	
45–54 years	92534	859	0.93	0.859–0.981	
55–60 years	2909	12	0.41	0.179–0.643	
*Number of donations*					

1	657886	3760	0.57	0.550–0.586	*χ * ^2^ = 842.45, *p* < 0.001
≥2	334760	556	0.17	0.152–0.180	
*Education level*					

Postgraduate	14912	17	0.11	0.060–0.168	*χ * ^2^ = 1964.844, *p* < 0.001
Bachelor	219580	246	0.11	0.098–0.126	
College degree	214390	497	0.23	0.211–0.252	
High school	153916	905	0.59	0.547–0.623	
Junior high school	139814	1305	0.93	0.875–0.975	
Primary education	9853	154	1.56	1.298–1.780	
Not defined	240181	1192	0.50	0.466–0.522	

**Table 2:** The multivariate logistic regression analysis for predictors of TP seropositivity.

	Sig	Exp (B)	95% C.I. for EXP (B)	
			Lower	Upper
Age	<0.001	1.499	1.448	1.552
Gender	<0.001	1.160	1.079	1.247
Education level	<0.001			
Primary education	<0.001	2.111	1.648	2.704
Junior high school	<0.001	1.824	1.637	2.032
College degree	<0.001	0.457	0.403	0.518
Bachelor	<0.001	0.292	0.250	0.340
Postgraduate	<0.001	0.154	0.092	0.257
Not defined	<0.001	0.230	0.208	0.254
Number of donations	<0.001	0.058	0.053	0.065
Constant	<0.001	2.920		

## Data Availability

The blood donors data presented in this study were collected and available from the database of blood management information system at the Blood Center of Zhejiang Province, China.

## Abbreviations

ALT: Alanine aminotransferase
ELISA: Enzyme-linked immunosorbent assay
Hb: Hemoglobin
HBsAg: Hepatitis B surface antigen
HIV: Human immunodeficiency virus
TP: *Treponema pallidum*
TPPA: *Treponema pallidum* particle agglutination
STDs: Sexually transmitted diseases
WHO: World Health Organization.
